# Verbal estimation of the magnitude of time, number, and length

**DOI:** 10.1007/s00426-020-01456-4

**Published:** 2020-12-17

**Authors:** R. S. Ogden, F. R. Simmons, J. H. Wearden

**Affiliations:** 1 Liverpool John Moores University, Liverpool, L33AF UK; 2 University of Keele, Staffordshire, ST5 5BG UK; 3 University of Manchester, Manchester, M13 9PL UK

## Abstract

Performance similarities on tasks requiring the processing of different domains of magnitude (e.g. time, numerosity, and length) have led to the suggestion that humans possess a common processing system for all domains of magnitude (Bueti and Walsh in Philos Trans R Soc B 364:1831–1840, 2009). In light of this, the current study examined whether Wearden’s (Timing Time Percept 3:223–245, 2015) model of the verbal estimation of duration could be applied to verbal estimates of numerosity and length. Students (*n* = 23) verbally estimated the duration, number, or physical length of items presented in visual displays. Analysis of the mean verbal estimates indicated the data were typical of that found in other studies. Analysis of the frequency of individual verbal estimates produced suggested that the verbal responses were highly quantized for duration and length: that is, only a small number of estimates were used. Responses were also quantized for number but to a lesser degree. The data were modelled using Wearden’s (2015) account of verbal estimation performance, which simulates quantization effects, and good fits could be obtained providing that stimulus durations were scaled as proportions (0.75, 1.06, and 0.92 for duration, number, and length, respectively) of their real magnitudes. The results suggest that despite previous reports of similarities in the processing of magnitude, there appear to be differences in the way in which the underlying representations of the magnitudes are scaled and then transformed into verbal outputs.

## Introduction

Successful interaction with the world requires accurate estimation of quantity in the environment: how many, how big, and how long. The ability to estimate numerosity, physical extent, and duration is therefore critical to human and animal survival. Although estimation of quantity is ubiquitous during daily life, little is known about how verbal estimation of quantity is accomplished (see Wearden, 2015, for discussion). This is in part because research has instead focused on how judgements of smaller and larger, shorter and longer, or less and more are performed. This paper therefore aims to further our understanding of how different domains of magnitude are estimated. In it we compare verbal estimates of numerosity, physical length, and duration and apply a recently developed model of verbal estimation of duration (Wearden, 2015) to the verbal estimation of numerosity and physical length. In doing so, we will establish whether there is support for a common processing system for magnitude estimation.

### Magnitude processing

Evidence from multiple sources suggests that the processing of different magnitudes draws on common resources. From a young age children are able to judge different domains of magnitude comparably, perhaps indicating a shared developmental trajectory (e.g. Droit-Volet, Clément, & Fayol, 2003; Droit-Volet, Tourret, & Wearden, 2004; Feigenson, 2007). In adults, interference studies consistently show that when simultaneously processing multiple magnitudes of different domains, performance is impaired in comparison with single magnitude processing (e.g. Coull, Charras, Donadieu, Droit-Volet, & Vidal, 2015; Dormal, Seron, & Pesenti, 2006; Oliveri et al., 2008; Xuan, Zhang, He, & Chen, 2007). In addition, comparable to the spatial–numerical association of response codes (SNARC) evoked by numerical stimuli, spatial-quantity association of response codes (SQARC) has been observed for stimuli varying in physical extent and duration (e.g. Simmons, Gallagher-Mitchell & Ogden, 2019). SNARC and SQARC effects are seen as evidence that number and, more recently, quantities, in general, are represented on an internal directional spatial continuum in which “few” is represented on the left and “many” is represented on the right. The presence of such effects for quantities of multiple magnitude dimensions suggests common spatial mapping.

Neuroimaging studies also suggest common neural resources for the processing of different magnitudes. Parietal activation is observed during the processing of number (Dehaene, Piazza, Pinel, & Cohen, 2003), duration (Pouthas et al., 2005), and space/length (Pinel, Piazza, Le Bihan, & Dehaene, 2004). There is also limited evidence that activation in the superior parietal lobule and the intraparietal sulcus is overlapping when processing numerosity and spatial extent (Kaufmann, et al., 2005). Furthermore, rTMS studies show that stimulation over the right intraparietal sulcus impairs the discrimination of numerosity and length (e.g. Dormal, Andres & Pesenti, 2012); however, this evidence is less clear for duration processing (e.g. Dormal, Andres & Pesenti, 2008).

Together, these behavioural and neural similarities have led to the suggestion that all magnitudes may share a common processing system. This idea was first suggested by Meck and Church (1983) after noting similarities between the responding of rats trained to discriminate number and duration. More recently, a theory of magnitude (ATOM) has been proposed (see Bueti & Walsh, 2009, and Walsh, 2003, for discussion). ATOM combines behavioural, developmental, and neuroimaging evidence to suggest that there is a common neural processing system for magnitude judgements located in the parietal cortex.

Although shared processing models are increasingly popular, there is also evidence of differences in performance when processing different types of magnitude. For example, Wearden, Parry, and Stamp (2002) compared memory for the duration of a line-like stimulus with memory of its length. Even in a situation where the stimuli judged were on average physically identical, the duration memory showed subjective shortening (Wearden & Ferrara, 1993), the effect that remembered duration seems progressively shorter with increasing retention interval. This was not true of length, which showed “normal” forgetting, i.e. progressively worse performance with increasing retention interval. Furthermore, interference studies in children (e.g. Droit-Volet et al., 2003) and adults (e.g. Casasanto & Boroditsky, 2008) often show asymmetrical patterns of interference when simultaneously processing multiple magnitudes. That is, one domain of magnitude, typically duration processing, appears more vulnerable to interference than other domains of magnitude. The processing of time, number, and length also appears to differentially recruit working memory and executive resources with temporal perception being more demanding of these resources than number or length (Ogden, Samuels, Simmons, Wearden, & Montgomery, 2017). One suggestion is that these differences may reflect the sequential nature of duration in comparison with the typically non-sequential nature of number and length (Droit-Volet, 2010; Ogden et al., 2017). However, an alternative possibility is that they may reflect differences in the way in which verbal labels are applied to quantity in different magnitude domains.

### Verbal estimation of magnitude

The verbal estimation of magnitude requires participants to assign verbal labels to stimulus properties such as size, physical length, duration, and numerosity. Verbal estimation is a commonly used task in the field of temporal perception, having been used to examine the perception of the duration of auditory (Wearden, Edwards, Fakhri, & Percival, 1998), visual (Wearden, Todd, & Jones, 2006), tactile (Jones & Ogden, 2016), and emotion-provoking stimuli (Gil & Droit-Volet, 2012) to name just a few. However, studies of the verbal estimation of other domains of magnitude (e.g. numerosity and length) are rarer.

Crollen, Grade, Pesenti, and Dormal’s (2013) comparison of the verbal estimation of time, number, and length suggests however that there may be fundamental differences in the way in which different domains of magnitude are estimated. Crollen, Grade, Pesenti, and Dormal (2013) required participants to make judgements of number (21–98 white dots), length [21- to 98-mm white rectangles (the original article gives lengths in cm, but this exceeds the size of normal computer monitors at the higher values, so seems likely to be an error)], and time (210–980 ms presentations of a 16-cm white dot). On average judgements underestimated real magnitude. The error rate, based on the percentage deviation from the target, with negative values representing underestimates and positive values overestimates was greatest for length (− 42.20), then for number (− 32.40), and smallest for time (−30.60). However, unlike most other studies of estimation, Crollen et al. (2013) used a potentiometer to produce a displayed number rather than asking participants to respond verbally. This may affect the way in which participants applied labels to the stimuli, in particular, the process of quantization of responses.

Unlike other tasks commonly used when assessing magnitude processing, verbal estimation is affected by a process called quantization of responses. Quantization is the tendency to use some estimates much more frequently than others (see Wearden, 2015 for discussion). For example, when estimating durations of less than 1 s, participants preferentially report estimates of 100, 200, and 500 ms, rather than for example, 128 ms, 232 ms, or 564 ms. Thus, quantization reveals the way in which raw representations of duration are expressed as common units of measurement. Comparing quantization effects across different magnitude domains may therefore reveal differences in the way in which the raw representations of these durations are converted into and expressed as common measurement units.

Wearden (2015) developed a model of verbal estimation performance which simulates this quantization process. The details are given later, but, in essence, the model converts a “raw” representation of a stimulus duration, or other magnitude which can vary continuously, into a small number of estimates which are output as behaviour, by virtue of a decision process. In the original 2015 article, all the results came from simulations, so the quantization model has not actually been fitted to experimental data from any study of duration estimates. This paper therefore aims not only to apply Wearden’s (2015) model to experimental data of duration estimates, but also to experimental data of numerosity and physical length estimates. In doing so, the paper will establish whether a common model can be used to explain the verbal estimation of quantity across different domains of magnitude.

### The current study

The current study sought to establish whether domain-based differences exist in the process of quantity estimation. Specifically, the study aimed to test whether comparable verbal labels are applied during the estimation of different domains of magnitude (duration, numerosity, and physical length). Furthermore, this study examined whether Wearden’s (2015) model of verbal estimation for duration could also model verbal estimation of numerosity and physical length.

Participants completed three separate verbal estimation tasks, one requiring estimation of the number of dots presented on the screen, one requiring estimation of the length of a line presented on the screen, and one requiring estimation of the duration of presentation of a square on the screen. Data from the three tasks were then compared in terms of mean estimates, estimate accuracy, and estimate variability. In addition, the pattern of quantization for each modality was explored. Finally, Wearden’s (2015) model was applied to each modality, and the model fit examined.

By comparing the patterns of quantization (number and frequency of verbal labels), and applying the model of verbal estimation, we will be able to establish whether the same underlying processes are involved in the verbal estimation of different quantities. If performance (comparable mean estimates, accuracy, and variability), quantization, and model fit are comparable across the duration, number, and length tasks, this would favour a shared resource account of magnitude processing (e.g. ATOM). However, if there are systematic differences in estimation performance and quantization, or if the model is unable to fit some domains or requires different parameters across the domains, this would suggest there may be underlying differences in way in which different magnitude domains are represented, processed, and labelled.

## Method

### Participants

Twenty-three Liverpool John Moores University students (mean age 18.91 years, SD 0.90, 4 males) participated in exchange for course credit. Credit was not contingent on performance. The study was approved by the Liverpool John Moores University Research Ethics Committee, and all participants gave informed consent. Participant numbers were chosen on the basis of a review of comparable published studies (e.g. Gil & Droit-Volet, 2012; Kanai, Lloyd, Bueti & Walsh, 2011; Pouthas et al., 2005; Wearden, Edwards, Fakhri, & Percival, 1998).

### Apparatus and materials

An IBM compatible computer running Microsoft Windows and a 17″ LCD monitor were used to present and record experimental events. Stimulus presentation and recording of keyboard responses were controlled via E-Prime version 2.0 (Psychology Software Tools, Inc., Pittsburgh, PA). The stimuli were developed using Microsoft Powerpoint.

### Experimental stimuli

#### Duration estimation

The to-be-timed stimulus was a black square 100 mm (11.42°) by 100 mm (11.42°) displayed in the centre of a white background. Eight different presentation durations were used: 150, 260, 350, 440, 560, 670, 760, and 850 ms.

#### Number estimation

The to-be-estimated stimuli were black circles 5 mm diameter in size presented for 1500 ms on a white background. The circles were randomly positioned on a 20 cm (22.62°) by 20 cm (22.62°) square grid of 100 possible locations around the centre of the computer screen. Eight different quantities of circles were presented: 15, 26, 35, 44, 56, 67, 76, and 85.

#### Line estimation

The stimulus was a black line presented for 1500 ms. The line was randomly positioned on a 20 cm by 20 cm square grid of 100 possible locations around the centre of the computer screen. Eight different lengths of line were presented: 15 (1.72°), 26 (2.98°), 35 (4.01°), 44 (5.04°), 56 (6.41°), 67 (7.76°), 76 (8.69°), and 85 (9.72°) mm, and all lines were 1 mm (0.11°) thick.

### Procedure

Participants were seated 50 cm from the computer screen. Participants then performed a duration estimation task, a number estimation task, and a length estimation task. Task order was randomised for each participant. Participants were not required to type the unit of measurement (e.g. ms or mm) with their estimate.

#### Duration estimation

Participants were informed that they would be presented with an image on the screen and that their task was to judge how long the image was presented for. Following stimulus presentation, a delay, the duration of which was drawn from a uniform distribution ranging from 500 to 750 ms, was interposed. Participants were then instructed to type their estimate of the square’s presentation duration using the keyboard. They were reminded that the maximum presentation duration was 1000 ms, no minimum presentation duration was provided, and estimates above 1000 ms were prevented by the experimental programme. Participants then pressed the spacebar to receive the next trial. Each of the eight presentation durations was used eight times during the experiment, giving a total of 64 trials. Trials were presented in a random order, and no performance feedback was given. The task took approximately 10 min to complete.

#### Number estimation

The procedure was identical to time estimation except for the following details. Participants were informed that they would be presented with images of dots and that their task was to estimate the number of dots presented. Participants were instructed that the maximum number of dots presented would be 100, no minimum quantity was provided, and estimates above 100 were prevented by the experimental programme.

#### Length estimation

All procedural details were the same as for time and number except for the following details. Participants were informed that they would be presented with an image of a horizontal line and that their task was to estimate the length of the line in millimetres. Participants were informed that the maximum possible line length was 100 mm, no minimum quantity was provided, and estimates above 100 were prevented by the experimental programme.

### Compliance with ethical standards

Ethical approval for this study was granted by Liverpool John Moores University Research Ethics Committee.

### Data analysis strategy

**Fig. 1:**
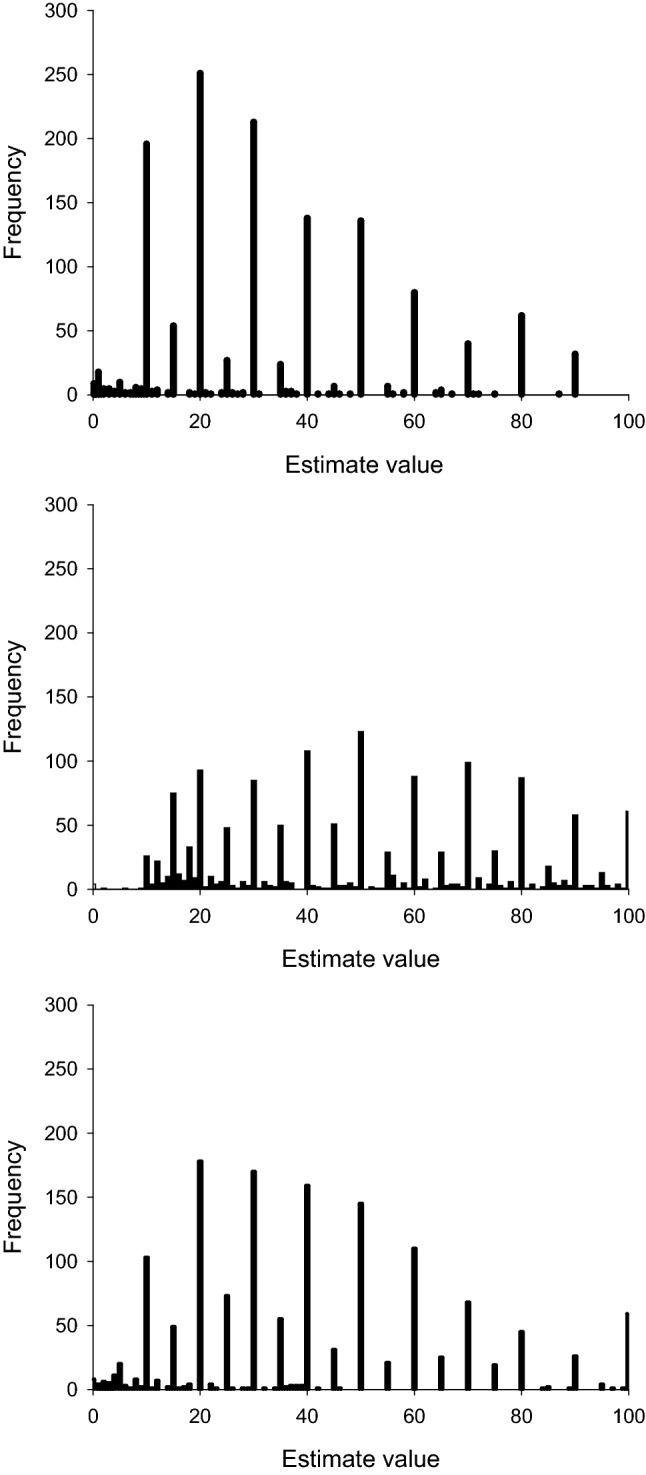
Absolute frequencies of estimate values used. Upper panel: time/10; centre panel: number; lowest panel: length

To make estimates comparable across the three conditions, estimates from the duration condition were transformed by dividing them by 10 prior to analysis. Quantization of responses was examined by calculating the frequency with which different estimates were emitted by participants (Fig. 1). To confirm that the data collected in this experiment were typical of those observed in other published studies, the mean verbal estimates, estimate variability, and estimate accuracy were compared for the duration, numerosity, and length conditions. In addition, rank correlations were performed to establish the relationship between performance across different magnitude domains. Finally, the model described in Wearden (2015) was applied to the data.

## Results

### Quantization of responses

Figure 1 shows the frequency of responses produced for time (upper panel), number (centre panel), and length (lowest panel), plotted against stimulus magnitude. It is immediately obvious that responses were highly “quantized”, that is, some estimates were used much more frequently than others. This quantization effect was particularly marked for time and length, but was also present for number. For time and length, around 10 values accounted for the vast majority of the estimates produced; in fact, the most frequent 10 estimates accounted for 83% and 78%, respectively, of all estimates for duration and length, but only 59% for number.

### Stimulus magnitude mean estimation

**Fig. 2:**
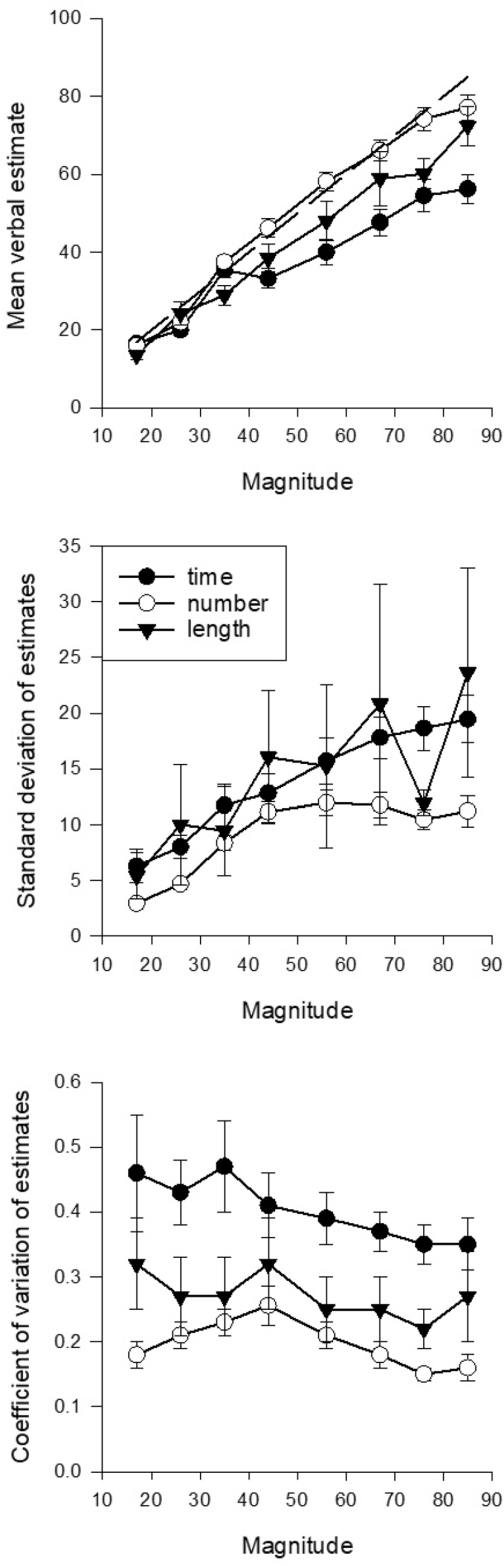
Upper panel: mean verbal estimates for time, number, and length as a function of magnitude. The dotted line shows accurate estimation. Centre panel: standard deviations of estimates for time, number, and length. Lowest panel: coefficient of variation (standard deviation/mean) of estimates of time, number, and length. Vertical lines show standard error of the mean. Magnitude values for time are all 1/10th of the real value in ms

The upper panel of Fig. 2 shows average magnitude estimates plotted against the stimulus magnitude for the three conditions (time, number, and length). Again, raw duration estimates were divided by 10. Examination of Fig. 2 suggests that, in all conditions, estimates increased approximately linearly with the magnitude of the stimulus. Estimates appeared longer in the number condition than the time and length conditions.

A repeated-measure ANOVA with within-subject factors of condition (time, number, length) and stimulus magnitude (15, 26, 35, 44, 56, 67, 76, 85) was conducted on the mean magnitude estimates. There was a significant main effect of condition *F*(2, 44) = 8.04, *p* = 0.001, $\eta_{{\text{p}}}^{2}$ = 0.27. Bonferroni post hoc tests confirmed significantly longer magnitude estimates in the number condition than the time condition (*p* < 0.001) but no significant differences between time and length (*p* = 0.24) and number and length (*p* = 0.23). There was significant effect of stimulus magnitude *F*(7, 154) = 187.67, *p* < 0.001, $\eta_{{\text{p}}}^{2}$ = 0.90. Inspection of the within-subjects contrasts showed that there was a significant linear effect *F*(1, 22) = 347.71, *p* < 0.001, $\eta_{{\text{p}}}^{2}$ = 0.94 and a significant quadratic trend *F*(1, 22) = 17.30, *p* < 0.001, $\eta_{{\text{p}}}^{2}$ = 0.44 for stimulus magnitude. There was also a significant interaction between stimulus magnitude and condition *F*(14, 308) = 4.68, *p* < 0.001, $\eta_{{\text{p}}}^{2}$ = 0.18. Within-subject contrasts again revealed a significant linear interaction *F*(1, 22) = 5.70, *p* = 0.03, $\eta_{{\text{p}}}^{2}$ = 0.21 and a significant quadratic trend *F*(1, 22) = 14.20, *p* < 0.001, $\eta_{{\text{p}}}^{2}$ = 0.39.

Linear regression of the average estimates for each stimulus duration for time, number, and length produced slopes of 0.58, 0.93, and 0.81, respectively, and intercepts of 8.60, 2.81, and 2.04. All slopes were significant (*p* < 0.001), but only the time intercept was (*p* < 0.05). *r*^2^ values were 0.95, 0.98, and 0.99, respectively. To further explore the interaction between condition and stimulus magnitude, individual linear regressions were conducted on each participant’s responses to provide slope and intercept values for each condition. A repeated-measure ANOVA conducted on the intercept values showed no significant difference in intercepts for the time (*M* 3.59, SD 7.43), number (*M* 2.00, SD 9.69), and length (*M* 3.22, SD 5.97) conditions *F*(2, 44) = 0.29, *p* = 0.75, $\eta_{{\text{p}}}^{2}$ = 0.01. The same analysis conducted on the slope values showed a significant effect of condition *F*(2, 44) = 9.20, *p* < 0.001, $\eta_{{\text{p}}}^{2}$ = 0.30. Slopes were significantly flatter in the time condition (*M* 0.65, SD 0.26) than the number condition (*M* 0.92, SD 0.22) (*p* < 0.001). There was no significant difference in the slopes of the time and length conditions (*M* 0.81, SD 0.33) (*p* = 0.08) or the length and number conditions (*p* = 0.39).

The centre panel of Fig. 2 shows the average standard deviations plotted against stimulus magnitude for each condition. Inspection of the figure suggests that standard deviations increased with stimulus magnitude, although it was less clear whether the different conditions produced different standard deviations. These suggestions were confirmed by statistical analysis. A repeated-measure ANOVA with within-subjects factors of condition and stimulus magnitude found a significant effect of stimulus magnitude *F*(7, 154) = 5.34, *p* < 0.001, $\eta_{{\text{p}}}^{2}$ = 0.20. Inspection of the within-subjects contrasts showed that there was also a significant linear effect *F*(1, 22) = 12.90, *p* = 0.002, $\eta_{{\text{p}}}^{2}$ = 0.37 and a significant quadratic trend *F*(1, 22) = 4.63, *p* = 0.04, $\eta_{{\text{p}}}^{2}$ = 0.17 for stimulus magnitude. However, there was no significant effect of condition *F*(2, 44) = 1.04, *p* = 0.36, $\eta_{{\text{p}}}^{2}$ = 0.05 nor any significant interaction between stimulus magnitude and condition *F*(14, 308) = 0.64, *p* = 0.83, $\eta_{{\text{p}}}^{2}$ = 0.03.

The lowest panel of Fig. 2 shows the mean coefficient of variation (CV: standard deviation/mean) plotted against stimulus magnitude for each condition. Inspection of the data suggests that coefficients of variation slightly declined with increasing stimulus magnitude, with values from number judgements being clearly lower than for duration or length.

A repeated-measure ANOVA with within-subjects factors of condition and stimulus magnitude found a significant effect of condition *F*(2, 44) = 14.83, *p* < 0.001, $\eta_{{\text{p}}}^{2}$ = 0.40. Bonferroni post hoc tests confirmed significantly larger CVs in the time condition (*M* 0.40, SD 0.24) than the number condition (*M* 0.20, SD 0.18) (*p* = 0.04) and length condition (*M* 0.27, SD 0.29) (*p* < 0.05) but no significant difference between number and length (*p* = 0.06). There was also a just significant effect of stimulus magnitude *F*(7, 154) = 2.08, *p* = 0.049, $\eta_{{\text{p}}}^{2}$ = 0.09. Inspection of the within-subjects contrasts showed that there was also a significant linear effect *F*(1, 22) = 5.71, *p* = 0.03, $\eta_{{\text{p}}}^{2}$ = 0.21. There was no significant interaction between stimulus magnitude and condition *F*(14, 308) = 0.64, *p* = 0.83, $\eta_{{\text{p}}}^{2}$ = 0.003.

### Estimate accuracy

**Fig. 3:**
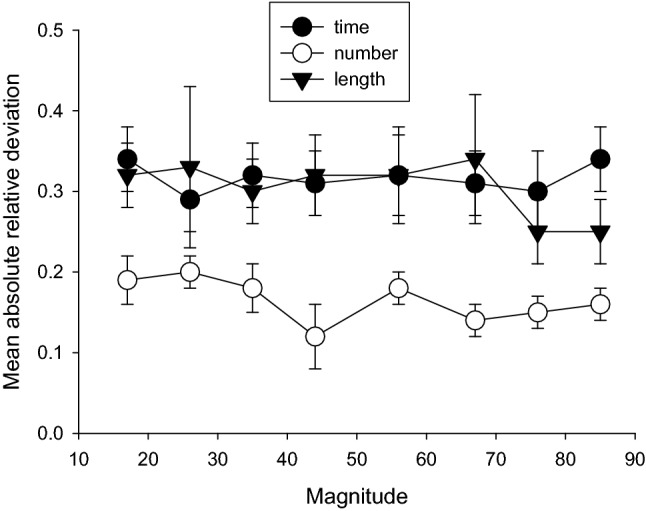
Mean absolute deviations for time/10, number, and length. Vertical lines show standard error of the mean

Figure 3 shows the mean absolute deviation between the estimate and the target. This was calculated by taking the absolute value of the deviation of the estimate from the target magnitude, and dividing by the target magnitude, for each participant and each stimulus value, and then averaging the resulting values. Inspection of the results suggests that accuracy was significantly better in the number condition than the time or the length conditions.

A repeated-measure ANOVA with within-subjects factors of condition and stimulus magnitude was conducted on the mean absolute deviation scores. There was a significant effect of condition *F*(2, 44) = 5.75, *p* = 0.006, $\eta_{{\text{p}}}^{2}$ = 0.21. Bonferroni post hoc tests confirmed significantly greater accuracy in the number condition than the time (*p* = 0.005) and length conditions (*p* = 0.02). Accuracy did not differ between the time and length conditions (*p* = 1.00). There was no significant effect of stimulus magnitude *F*(7, 154) = 0.75, *p* = 0.63, $\eta_{{\text{p}}}^{2}$ = 0.03 and no significant interaction between stimulus magnitude and condition *F*(14, 308) = 61, *p* = 0.85, $\eta_{{\text{p}}}^{2}$ = 0.03.

### The relationship between performance measures across magnitude domains

Spearman’s rank correlations were conducted to establish whether measures of estimation in one domain were related to the same measure of estimation in another domain. For example is number estimate accuracy related to time estimate accuracy. This analysis is shown in Table 1.

**Table 1:** Correlation coefficients for the relationships between measures of accuracy, CV, and number of verbal estimate values across the three domains of magnitude

	Time × number	Time × length	Number × length
Accuracy	0.13	0.26	− 0.09
Variability (CV)	0.36	0.23	0.44
Number of verbal estimate values	0.48	0.37	0.47

## Discussion and data modelling

This paper aimed to establish whether Wearden’s (2015) model of the verbal estimation of duration could be applied to other magnitude domains, in this instance estimates of numerosity and physical length. Examination of the quantization of responses suggests that there were notable differences in the way in which the raw representations of data were quantized for the three magnitudes. For time and length, the histograms showed that a relatively small number of values were frequent. For number however, flatter histograms indicated that a greater number of response values were produced by participants, and as a result, each was used less frequently. There therefore appear to be notable domain-based differences in the way in which raw representations of magnitude are expressed using conventional units of measurement.

The analysis of the mean verbal estimates, estimate variability, and estimate accuracy suggests that mean estimates all increased approximately linearly with real magnitude, but slopes differed, with slope from number and length being significantly higher than for duration. Coefficients of variation differed across magnitude domains, with number showing the smallest values. In terms of accuracy, number judgements clearly corresponded more closely to real magnitudes than did duration or length.

These findings contrast with those of Crollen et al. (2013) who found that accuracy in terms of deviation from the real magnitude was in a different order from ours. If our data are treated in the same way as theirs, the “error rates” for the three modalities were number, − 5.2, length, − 10.2, time, − 20.7, so all modalities involved underestimation, as in Crollen et al. (2013), although this was very slight in the case of number in our study. It is unclear why Crollen et al.’s study and our own produced such different results. There are a number of procedural differences between the studies: we used lines for length judgements rather than rectangles, a black square on a white background for duration, as opposed to a white circle on a black background for theirs. In addition, the method used for estimation was different in their study and involved people turning a potentiometer to produce a displayed number rather than giving the response verbally as in our experiment. However, given that the ranges of values for the different magnitudes were similar in the two studies, the difference between them remains somewhat mysterious. Indeed, their results for numerosity in particular are different not only from our results, but also that of others reviewed in the following paragraphs, which have generally found reasonably accurate judgements of numerosity, whereas Crollen et al. found numerosity judgements to be the least accurate.

Our data on number judgements are however generally consistent with those obtained previously, although results were found by very different methods. Numerical judgements without explicit counting are said to be based on an “approximate number system” (ANS), which humans may share with animals (Feigenson, Dehane & Spelke, 2004). Many studies of the ANS have focussed on acuity, that is, the smallest difference or ratio in two numerical quantities that can be discriminated, often within a developmental context. A common technique involves presenting participants with two side-by-side displays of items and asking which side had the larger number (see Mussolin, Nys, Leybaert & Content, 2016 for a review). Performance in studies examining non-verbal numerosity discrimination has consistently indicated that accuracy is ratio dependent with less accurate responses when the ratio is lower; there is also a developmental increase in acuity with age (Mussolin et al., 2016). The ratio-dependent nature of non-verbal quantity judgements is often interpreted as a reflection of increasingly noisy or fuzzy representations of larger numbers and quantities (Feigenson et al., 2004, however see Halberda & Odic 2015, for an alternative explanation in terms of the confidence of individual judgements). Both ratio dependency and developmental increases in acuity are mirrored in judgement tasks involving other magnitude domains including duration and spatial extent (Cantlon et al., 2009; Odic, Libertus, Feigenson, & Halberda, 2013). In the present study, performance on all three of the magnitude judgements (number, length, and duration) reflects scalar variability (i.e. increasing variability with increasing quantity) that is seen as a key signature of the ANS (Mussolin et al., 2016).

Although most studies investigating the ANS have utilised non-verbal quantity comparison, some earlier ones looked at something closer to scaling or judgements as a function of number. Whalen, Gallistel, and Gelman (1999) and Cordes, Gelman, and Gallistel (2001) used a method involving key pressing. An arabic numeral was shown (e.g. “7” or “25”, the smallest and largest values used), and the participant was required to press a key as fast as possible for the number of times indicated in the display. In Whalen et al.’s key press study the mean and standard deviation of the number of presses grew linearly with the number requirement, and the mean tracked the number requirement close to accurately, although inspection of the data (their Fig. 3, p. 133) suggests that the number of key presses overshot the number requirement for some participants. The coefficient of variation (standard deviation/mean) was nearly constant as the number requirement varied from 7 to 25, indicating conformity to the scalar property of variability often found in duration judgements (e.g. Wearden & Lejeune, 2008).

Cordes et al. (2001) performed a similar study, with more controls for explicit counting, but their condition most similar to that in Whalen et al. (1999) produced very similar results to the earlier study. As well as a key-press experiment, Whalen et al. also used a “flash-count” method, where between 7 and 25 irregularly spaced flashes were presented, and the participant was required to estimate the number verbally. Both the mean and standard deviation increased approximately linearly with the number requirement, although it seemed as if the estimates were more likely to underestimate the number rather than overestimate it, judging from their Fig. 4 (p. 135). The coefficient of variation was approximately constant, although for some participants appeared slightly lower and the longer number values. Both the key-press and flash-count methods have the potential problem that the time taken to make the response (key-press method) or the time taken for the display (flash count method) might be used to generate the response, but Whalen et al. (1999) argue convincingly that participants were probably not using time. This issue does not arise with our verbal estimation method, of course.

**Fig. 4:**
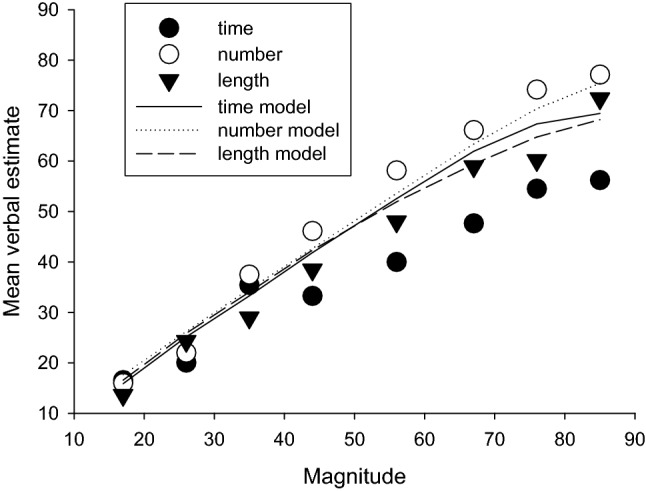
Mean verbal estimates for time/10, number, and length, and values from the Wearden (2015) model (lines), with all magnitudes veridical

Overall, results from Whalen et al. (1999) and Cordes et al. (2001) are similar to those reported in the present article. Average number estimates tracked the number requirement closely (upper panel of our Fig. 2), standard deviations increased with the number requirement (centre panel of Fig. 2), and the coefficient of variation did not change systematically with range of numbers presented (lowest panel of Fig. 2), even though the largest numbers we used were larger than any in Whalen et al. (1999) or Cordes et al. (2001). Considering the differences in methods between their studies and ours, the general consistency of results is noteworthy.

Results from our duration estimates were also similar to those found in previous studies, in showing a kind of “linear underestimation” of real duration by average estimates. The average slope value relating our mean estimates to real duration was 0.65, as mentioned earlier. To provide comparisons, we took data from several studies where verbal estimation of the duration of visual stimuli (usually squares of colour on a computer screen) had been used. The data came from Penton-Voak, Edwards, Percival, and Wearden, (1996), Fig. 3 (p. 315), range 123–863 ms, Wearden et al. (1998) Fig. 2 (p. 106), range 77–1183 ms, Wearden et al. (1998) Fig. 3 (p. 110), range 77–1183 ms, and Wearden et al. (2006) Fig. 3 (p. 1717), upper and lower panels, range 77–1183 ms. Slope values from regression of mean estimates against stimulus duration were, respectively, 0.76, 0.78, 0.66, 0.76, and 0.76. The implications of this consistent linear underestimation of duration will be discussed further below.

### Modelling the verbal estimation of magnitude

The principal problem for modelling verbal estimation is quantization of responses, which was present in our data very strongly for duration and length, but also present, albeit less strongly, for number. Such quantization means that algebraic models may struggle to capture important aspects of the data, but Wearden’s (2015) model was intended to simulate the quantization effect, so can be applied here. Although the model involves a considerable amount of calculation, in its initial form it is very simple in principle. When a stimulus of magnitude *s* is presented, this is transformed into a value s* which is randomly selected from a Gaussian distribution with a mean *s*, and some coefficient of variation, *c*. This internal representation is not, however, directly translated into estimates. Rather, a quantization process occurs. Suppose, as is the case in the simulation to be presented below, that only 10 outputs are allowed. Each of these outputs has a value (e.g. “100”, “50”), and also a weight, which determines how “attractive” this output value is. The attraction between any s* and any particular output value is determined by “distance” (absolute deviation) and also by the weight, and the “attractive power” of any particular output value is determined by the weight divided by the distance. The attractive power of all 10 output values is calculated for each s*, and the two strongest compete probabilistically. That is, they compete in terms of their weight/distance measure. For example, if this measure is twice as great for one output value than for the other, then it is twice as likely that the stronger one will be chosen as the estimate. A fuller account of the model and exploration of its properties are given in Wearden (2015).

The model might be best considered to reverse engineer the data it treats, as the output values and weights are derived from data. In the present case, the 10 most frequent output values were used for each condition, and the weights were determined by the relative frequency of each output. In the original operation of the model, the only fitting parameter was *c*, the coefficient of variation of the stimulus representations, as weights are calculated from the data, and the distance depends on the value of s*. Figure 4 shows the results when the coefficient of variation was 0.4 (as this value was close to values found in the best-fitting models to be discussed below). The first important result is that the original form of the model could not fit data from the different conditions: in particular, the marked difference between means for number and duration could not be accurately simulated. This failure of the model, when the scaling of all stimuli is kept constant (that is, all were represented by their physical values, on average), shows that the quantization, by itself, cannot produce the difference between duration, number, and length which we observed in our experiment. Something else is apparently needed. Given the “linear underestimation” of duration discussed earlier the simplest solution seemed to be to take this into account by rescaling.

**Fig. 5:**
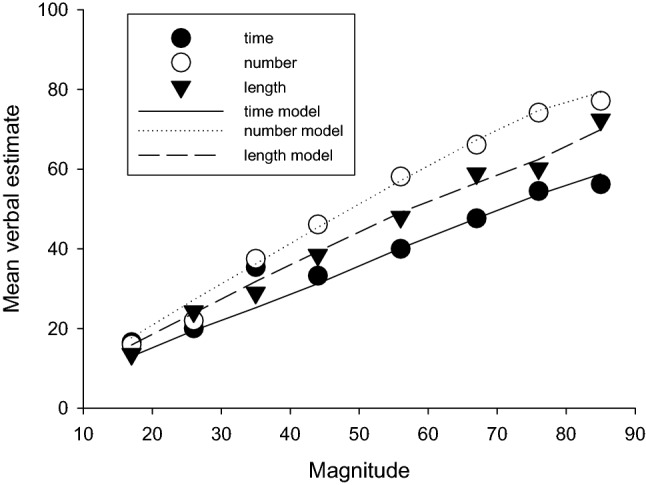
Mean verbal estimates for time/10, number, and length, and values from the Wearden (2015) model (lines). Magnitudes and linearly transformed (see text for details)

To do this, a scaling constant was used, that is, the real magnitudes were multiplied by a factor *k*, then the model operated as normal. We explored various k and c values with the model, and it became clear that a c value of approximately 0.4 was needed, and that the k value was modality-dependent. We used the approximate *k* and *c* parameters for each modality and then varied them in 0.01 steps to find the smallest absolute deviation between the mean estimates in data and the means resulting from the model. Figure 5 shows the results. The best-fitting *k* and *c* values were: duration, 0.75 and 0.4, number, 1.06 and 0.33, and length, 0.92 and 0.45. Figure 5 shows the resulting fits of the model.

Inspection of the results in Fig. 5 shows that the model fitted data well, with the mean estimates for duration, number, and length being closely modelled and appropriately spaced apart. The mean absolute deviations (sum of the absolute deviations between the model’s fit and data divided by the number of data points, 8) were duration, 0.8, number, 1.70, and length, 1.76. Given that the average magnitude value was 50.5, this implies an average deviation between 1.6 and 3.5%. As discussed in Wearden (2015) the quantization model can only fit approximately at best, as it does not take account of all the output values used (in the present case it only uses 59% of them for the number simulation) and in addition may have other deficiencies, such as too simple a decision rule. Nevertheless, it is clear that with the scaling parameter in use, the model can fit the mean data produced very well. If an intercept had been added as well as the scaling constant, the fit would most likely have been even better.

The scaling constants needed varied systematically with the stimulus type modelled. For number, the scaling produced average overestimation of the physical magnitude, whereas for duration and length a scaling that produced underestimation was needed, particularly for duration. We leave aside for a moment the question of where different output values come from and look instead at one of the consequences of scaling. Figure 1 shows the empirical quantization of data, with a small number of output values dominating, particularly for duration and length. We used the model to explore the effect of different sorts of scaling on quantization.

**Fig. 6:**
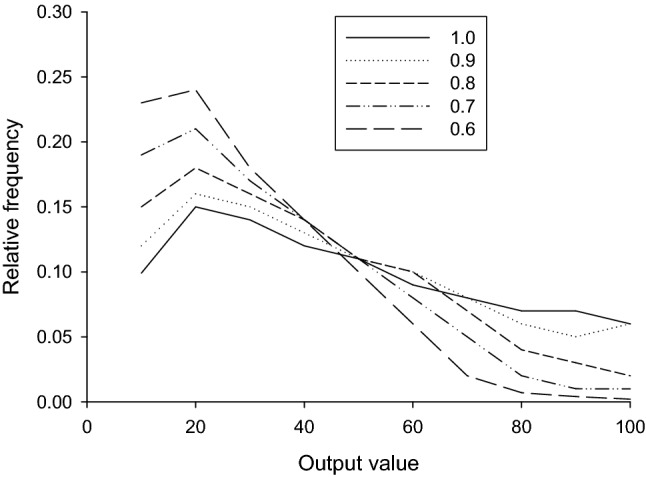
Effects of quantization and magnitude scaling on simulated output frequencies (see text for details)

To do this, we supposed that the only output values permitted were 10, 20, … 100, when the stimulus magnitudes were the same as in our experiment, and all values had the same weight, a relative frequency of 0.1. Next, we varied the scaling parameter over values from 1.0 (veridical representation) to 0.6. Results are shown in Fig. 6. As the scaling parameter declines in value, a number of the smallest output values come to dominate output. So, it seems possible that the different quantization weights found in data (Fig. 1) are a consequence of the different scaling needed to fit the estimates for the different experimental conditions. To put it another way, although quantization, by itself, cannot produce the difference in means between the conditions (as Fig. 4 shows), the very scaling needed to produce this difference in means will almost certainly influence the output value weights, changing them from the flattish profile shown by number (which has the scale value closest to one) to the highly skewed profile shown by duration (which needed to smallest scale value to fit data, as in Fig. 5). Note, in addition, that even with veridical representations of the stimulus magnitudes (scale = 1.0) and evenly spaced output values with equal weights, the resulting simulated behaviour does not have equally likely output values: the scalar representations of duration, which results in larger standard deviations at larger magnitudes, distort the underlying veridical representation in output, interacting with the actual magnitudes used, and results in some output values having around twice the relative frequency of others.

Although scaling can alter the obtained relative frequency of output values used, as Fig. 6 shows, it cannot be responsible for what the output values actually are. When estimating duration, for example, why do people tend to use “round” values such as 100 and 500 and hardly ever 650, let alone 479? We cannot provide a simple answer to this, but only some suggestions. One is that people have some impression of their ability to discriminate durations which is too pessimistic. For example, they may imagine that they can distinguish one second from half a second, but not 600 ms from 500 ms, whereas in certain procedures their ability to do this is much better than they believe (e.g. using a bisection or temporal generalization technique 500 and 600 ms can reliably result in different numbers of responses, see Wearden, 1991, 1992). If, as has been previously suggested, quantities are represented spatially on a mental “number” lines (e.g. Dehaene, 1992), it is possible that the quantization effects observed in this paper reflect differences in the way in which these lines are populated. The use of a small number of round values during duration estimation may suggest that the “duration line” is populated by a small number of sparsely spaced values. For number, in contrast, people know that the integer number line is completely filled, not least because of ages and dates. Anyone over 30 must once have been 19 or 27, although they will be less familiar with, for example, 19 or 27 cm, or 190 or 270 ms. People also have greater experience of providing precise numerical estimates for quantities than they do of providing precise estimates of length and duration. This experience, coupled with greater opportunities for feedback on the accuracy of their estimates, may have enabled people to use a wider range of output values for number judgements than for duration or length.

Another possibility is that quantization differences are influenced by the need to translate a continuous stimulus into a discontinuous output. Duration, for example, is a continuous dimension; however, the process of verbal estimation requires this continuous representation to be translated into a discontinuous verbal output. This translation process may affect the estimates produced, the estimate accuracy and the estimate variability, possibility by creating a greater reliance on fewer verbal values. In contrast, numerosity is a discontinuous dimension and therefore verbal estimation of this magnitude does not require translation. This may facilitate the use of a wider range of verbal outputs, resulting in less variability and increased estimate accuracy. Relatedly, numerosity is the only domain that does not require conversion into a conventional unit of measurement. It is therefore possible that the requirement to convert a raw representation of length into millimetres and duration in milliseconds contributed to the quantization effects observed. It is also possible, however, that the use of a larger stimulus range for duration estimates (0–1000 ms) encouraged a greater level of quantization than for number and length in which the range was smaller (0–100). Future research should therefore examine how duration ranges influence the quantization process.

Finally, we should discuss the issue of “linear underestimation” for duration, which seems at first sight to contradict the principle of scalar timing theory that duration representations are on average accurate (Wearden & Lejeune, 2008). Most commonly used timing tasks, such as bisection and temporal generalization (see Wearden, 2016, for discussion), involve relative rather than absolute timing. Here, stimuli to be judged are compared with previously presented standards, so if all are linearly underestimated (that is, they would be judged as *x*% of their real time value in a verbal estimation task), performance would be unaffected because of the scale invariance required by scalar timing theory. The same even applies to the more complex fractionation task employed by Wearden and Jones (2007), where people are asked what fraction one duration is of another one. Performance on these relative timing tasks would be the same whether or not the durations involved in them would be estimated accurately in terms of real clock-measured duration, as long as they were linearly scaled. One task that does seem to contradict the idea of linear underestimation of time is interval production with feedback, as in Wearden and McShane (1988). Here, people are given a target time and repeatedly produce it with feedback after each production. The average time produced tracks real time almost perfectly, suggesting accurate underlying timing. However, as Wearden and Jones (2007) point out, interval production with feedback tells us nothing about the timescale underlying performance. So, linear underestimation is not incompatible with performance on the timing tasks actually studied, although the question of why it arises obviously remains an open one.

The overall relation between estimate accuracy and variability found when comparing judgements of number with those of the other domains is in accord with work by Nash (2017), although this takes a very different approach from the one used in the present article. However, if we examine the present data, we see that judgements of number deviate on average less from accuracy than do judgements of time and length (upper panel of Fig. 2) and in addition are less variable both absolutely (centre panel of Fig. 2) and in terms of variation around their mean (lower panel of Fig. 2) in accord with Nash’s ideas.

One obvious remaining question is whether the domain-based differences in scaling and underestimation, observed in the current study, would persist if the stimuli were presented sequentially. Droit-Volet (2010) observed that the similarity between performance on time, number, and length bisection increased when the stimuli were presented sequentially rather than non-sequentially. Whilst this may suggest that sequential presentation may have resulted in the scales, and thus the responses, being more similar across the three domains, this conclusion may be premature. Droit-Volet’s (2010) results are based on bisection performance, in which perceived magnitudes are only categorised as short/long or few/many. This is unlike in the verbal estimation method used in this study in which numerical values are used to quantify the stimulus magnitude. It is presently unclear how sequential verbal estimation of time or indeed any other magnitude is accomplished. One possibility is that verbal labels would be applied to each “piece” of the sequential presentation and then these would be “added up” to form a magnitude estimate. If this were the case, we speculate that the scaling differences observed in the current study would persist as we have no reason to believe that smaller units of magnitude would be scaled differently to larger ones used in non-sequential presentation. However, we also acknowledge that is possible that the additional cognitive load associated with applying a verbal label to a sequentially presented stimuli may introduce additional variances which may be unique to one magnitude or general across all magnitudes. Further research should therefore establish how sequentially influences the coding of raw magnitude representations into verbal labels.

In conclusion, this study shows that despite growing evidence of behavioural and neural similarities in the way in which different domains of magnitude are processed, there are systematic differences in the way in which the underlying representations of the magnitudes are scaled and then transformed into verbal outputs. This is evidenced by a number of factors, Firstly, by the differing use of verbal outputs for the different domains of magnitude, as seen in Fig. 1. Secondly, domain-based differences in the accuracy and variability of estimates. Thirdly, domain-based differences in the scaling constant required to fit Wearden’s (2015) verbal estimation model to the data. Although it is presently unclear as to why different domains of magnitudes are scaled and quantized differently, it seems that mean measures of behaviour can be accurately simulated only if these differences are taken into account.
